# Effects of an Interchain Disulfide Bond on Tropomyosin Structure: A Molecular Dynamics Study

**DOI:** 10.3390/ijms19113376

**Published:** 2018-10-28

**Authors:** Natalia A. Koubassova, Sergey Y. Bershitsky, Andrey K. Tsaturyan

**Affiliations:** 1 Institute of Mechanics, Moscow University, 1 Mitchurinsky prosp., 119234 Moscow, Russia; tsat@imec.msu.ru; 2 Institute of Immunology and Physiology, Ural Branch of Russian Academy of Sciences, 106 Pervomayskaya ul., 620049 Yekaterinburg, Russia; s.bershitsky@iip.uran.ru

**Keywords:** tropomyosin, disulfide bond, molecular dynamics, bending stiffness, hydrogen bonds

## Abstract

Tropomyosin (Tpm) is a coiled-coil actin-binding dimer protein that participates in the regulation of muscle contraction. Both Tpm chains contain Cys190 residues which are normally in the reduced state, but form an interchain disulfide bond in failing heart. Changes in structural and functional properties of Tpm and its complexes with actin upon disulfide cross-linking were studied using various experimental methods. To understand the molecular mechanism underlying these changes and to reveal the possible mechanism of the involvement of the cross-linking in heart failure, molecular dynamics (MD) simulations of the middle part of Tpm were performed in cross-linked and reduced states. The cross-linking increased bending stiffness of Tpm assessed from MD trajectories at 27 °C in agreement with previous experimental observations. However, at 40 °C, the cross-linking caused a decrease in Tpm stiffness and a significant reduction in the number of main chain hydrogen bonds in the vicinity of residues 133 and 134. These data are in line with observations showing enhanced thermal unfolding of the least stable part of Tpm at 30–40 °C and accelerated trypsin cleavage at residue 133 at 40 °C (but not at 27 °C) upon cross-linking. These results allow us to speculate about the possible mechanism of involvement of Tpm cross-linking to heart failure pathogenesis.

## 1. Introduction

Tropomyosin (Tpm) is a coiled-coil protein that consists of two parallel α-helical polypeptide chains. The N- and C-terminal parts of the Tpm molecules bind each other in a “head-to-tail” manner via so-called overlap junctions to form a semi-rigid strand that binds the helical groove on the surface of an actin filament [1,2,3]. In striated muscle, Tpm participates in the regulation of contraction by controlling access of myosin heads to actin [4,5,6]. Mathematical modeling suggests that high cooperativity of Ca^2+^ regulation of muscle contraction requires high stiffness of the Tpm strand. The high stiffness is necessary for transmitting azimuthal Tpm displacement with respect to the axis of the actin filament to adjacent Tpm molecules to open or close neighbor actin sites for myosin binding [7,8,9].

A cysteine residue C190 is present in all human striated muscle Tpm isoforms. Under oxidizing conditions, C190 residues of two α-helices form a disulfide bond [10]. Although the vast majority of the SH- or thiol groups of the C190 residues are in a reduced state in both skeletal [11] and cardiac [12] muscles, a substantial fraction of interchain disulfide bonds was found in the myocardium of experimental animals subjected to microembolization [13] and of patients with end-stage heart failure [14]. These data led to a hypothesis that the interchain cross-linking can be involved in the pathogenesis of myocardial dysfunction [13,14].

The effect of the disulfide cross-linking on the structural properties of Tpm was studied 40 years ago by Lehrer using fluorescence and circular dichroism measurements [15]. He found that the cross-linking significantly stabilized the Tpm structure against thermal or guanidine hydrochloride induced denaturation. At the same time, the cross-linking caused a destabilizing pre-transition at a range of 30–45 °C that involves 10–20% of the total length of the Tpm α-helices. Differential scanning calorimetry (DSC) studies have also revealed a shift in the major thermal transition from ~45 to 59 °C and the appearance of a “pre-transition” melting peak in the 25–40 °C region [16,17]. Further DSC studies with careful deconvolution and analysis of the calorimetric domains have shown that the increase in thermal stability mainly involves the C-terminal part of Tpm, with no appreciable changes in the stability of its N-terminal part [18,19]. As for the effect of the cross-linking on the least thermostable domain of Tpm, presumably corresponding to the middle part of Tpm [19], the results of these experiments were somewhat controversial. An increase in enthalpy of this domain upon cross-linking was observed in the presence of cardiomyopathy-associated mutations D175N and E180G, but not for the wild type (WT) Tpm [18]. On the other hand, in recent studies, a shift of the transition towards lower temperature and an increase in the enthalpy associated with this peak were detected for WT Tpm [19] in agreement with earlier optical measurements [15]. Data showing that at 26 °C the cross-linking did not affect the rate of trypsin cleavage of Tpm, while it slightly increased the rate at 40 °C [20] are in line with the DSC data. The primary site of Tpm trypsin cleavage is between residues R133 and A134 located in the least stable part of Tpm [2]. Therefore, enhanced thermal disorder of this region upon the cross-linking is expected to accelerate trypsin cleavage of Tpm.

Interchain cross-linking was reported to decrease the affinity of Tpm for actin [21,22] and the inhibitory effect of Tpm on the actin-myosin ATPase [23]. The effects of the cross-linking on the functional and regulatory properties of Tpm and its complexes with actin, myosin and Ca^2+^-binding regulatory protein, troponin (Tn), were studied recently [22]. Despite an increase in the global thermal stability of Tpm [16,17], cross-linking shifted the temperature-induced Tpm dissociation from actin towards lower temperatures and significantly increased the sliding velocity of reconstructed regulated thin filaments (containing F-actin, Tpm, and Tn) at a saturating Ca^2+^ concentration in vitro [22]. The increase in the sliding velocity was especially pronounced if both myosin and Tn were from cardiac, not skeletal muscle [22]. In addition, the cross-linking caused a 1.44-fold increase in the bending stiffness of the actin-Tpm filaments measured with a two-beam optical trap [22].

To understand the possible molecular mechanisms underlying the above-listed changes in structural and functional properties of Tpm caused by the interchain cross-linking, we performed molecular dynamics (MD) simulations of the central part of the Tpm in both cross-linked and reduced states. In particular, we were interested in the mechanism of long-range effect of the cross-linking that increases the overall stiffness of Tpm and decreases thermal stability of the least thermostable middle part of the molecule, although it increases the stability of the C-terminal part of Tpm near to Cys190. MD simulations were performed at 27 and 40 °C to reveal possible reasons for these apparently contradictory effects of temperature on structural properties of Tpm.

Obtaining Tpm in the fully reduced state is time-consuming. Moreover, even then, spontaneous cross-linking can occur during storage of frozen protein. For these reasons, some authors (see, for example, [24,25]) use the C190A mutant of Tpm (where the Cys190 residue is substituted for Ala) as an analog of Tpm in the reduced state. Although some properties of the C190A Tpm were shown to be similar to those of WT Tpm in the reduced state [24], we also performed MD simulation of the C190A Tpm mutant to compare its dynamic properties with those obtained for WT Tpm in the cross-linked and uncross-linked states.

## 2. Results

### 2.1. Disulfide Cross-Linking Causes an Increase in Bending Stiffness of Tpm

**Figure 1:**
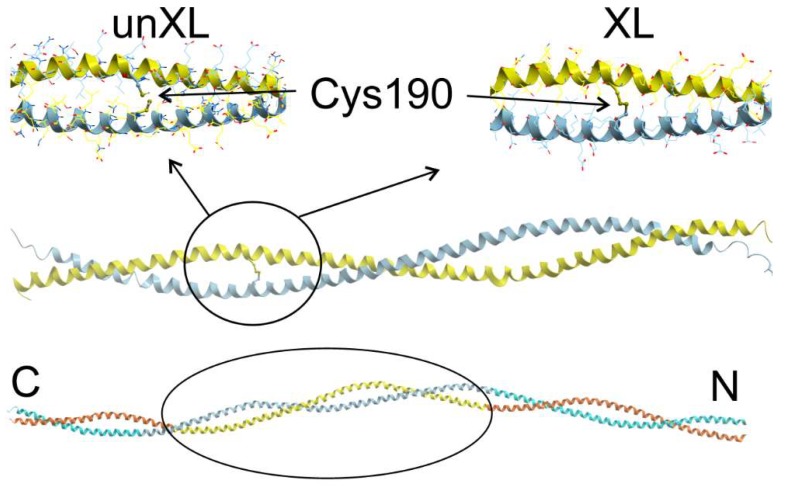
Close view on the snapshots of the MD trajectories in the vicinity of the Cys190 residues for uncross-linked (un-XL) and cross-linked (XL) Tpm are shown in top insets. The middle part of Tpm used for MD simulations is shown in the middle. The position of the middle part of Tpm used for MD simulations in whole Tpm molecule is shown in the bottom with marked N- and C-terminals. The figure was prepared with ICM-Browser Pro v. 3.8-7 (MolSoft, LLC, La Jolla, CA, USA).

Models used for MD simulations are shown in Figure 1. A crystal structure of the middle part of Tpm [26] (PDB code 2B9C) with a disulfide bond between two Cys190 residues was used as the starting model of cross-linked Tpm. Models of uncross-linked Tpm and the C190A Tpm mutant were prepared from the 2B9C structure with UCSF CHIMERA package [27]. Details of MD simulation and methods used for analysis of the MD trajectories are described in Section 4.

**Figure 2:**
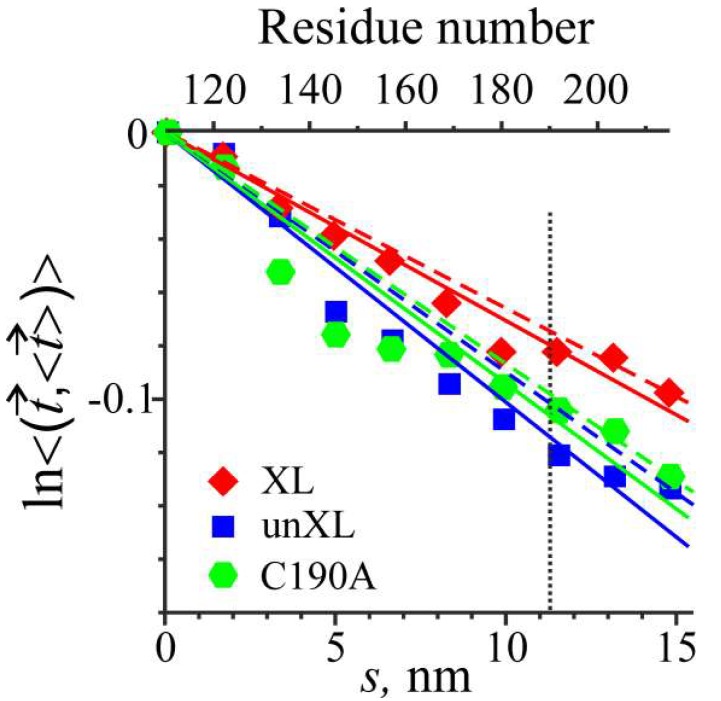
Flexibility plots used for estimation of persistence length of the middle part of Tpm segments MD trajectories at 27 °C. Logarithm of the time-averaged scalar product of the unit tangent vector $\vec{t}$ and the time-averaged unit tangent vector $\langle \vec{t}\rangle$ is plotted against the distance along the axis of the Tpm coiled-coil. Blue squares, red diamonds and green hexagons correspond to uncross-linked, cross-linked and C190A Tpm, respectively. The solid lines show the slope of least squares fit; the dashed lines are the secants connecting the first and the last points; and the dotted vertical line shows the position of residue 190. The bending stiffness is proportional to persistence length and reciprocal to the slopes of the lines. S.E.M. bars for each data point are within data symbols. See text and Table 1 for details of the flexibility analysis.

The data used for the estimation of persistence length of Tpm from MD trajectories are shown in Figure 2. The analysis of the bending stiffness of the middle part of Tpm molecule was performed with a method initially suggested by Li et al. [28] and later modified for a more precise description of the flexibility of intrinsically curved coiled-coils [29].

The MD simulations show that the middle parts of both uncross-linked Tpm and the C190A Tpm mutant are less stiff than that of cross-linked Tpm (Figure 2). Two different approaches for stiffness measurement using either least squares fit or secant give similar results (Figure 2 and Table 1). The total increase in the bending stiffness of Tpm molecules caused by the cross-linking was 37–44% (Table 1). The C190A mutation did not induce significant changes in Tpm bending stiffness compared to uncross-linked Tpm (Figure 2). The most flexible region can be detected from the local deviation of local slope of the plots shown in Figure 2 from straight lines. For the C190A Tpm mutant and uncross-linked Tpm, this most flexible Tpm region was located between residues 125 and 140. Stiffness distribution along the middle part for cross-linked Tpm was more uniform (Figure 2).

The time-average numbers of hydrogen bonds (H-bonds) within the main chain in different regions of the middle part of Tpm are shown in Table 1. As trypsin cleavage requires access of the active center of the enzyme to the peptide bond between residues R133 and A134, we also calculated the number of H-bonds within the main chain residues 129–138, i.e., in close vicinity of the trypsin cleavage site.

A significant increase in bending stiffness at 27 °C upon interchain cross-linking was not accompanied by an increase in occupancy of the H-bonds holding Tpm α-helices. The cross-linking rather led to a small and insignificant decrease in the number of main chain H-bonds in the middle part of Tpm (Table 1).

### 2.2. Effects of Interchain Cross-Linking on Tpm Stiffness and Stability Are Temperature Dependent

**Figure 3:**
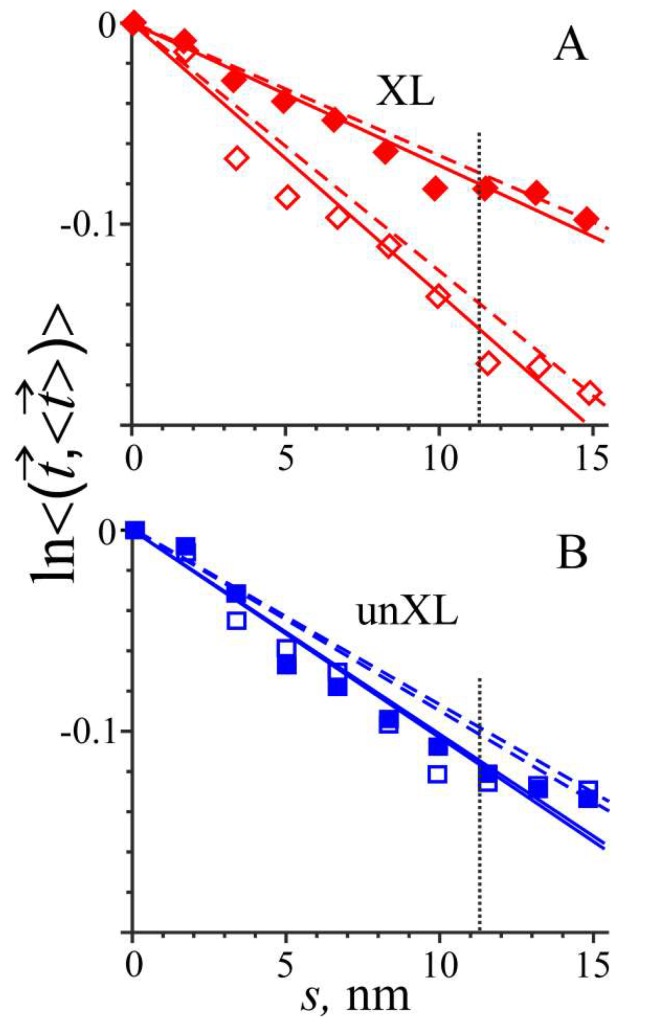
The flexibility plots obtained from MD simulations of cross-linked (XL) (**A**) and uncross-linked (unXL) (**B**) Tpm at 27 °C (closed symbols) and 40 °C (open symbols). Same notations are used for the straight lines as in Figure 2.

An increase in temperature from 27 to 40 °C in MD simulation of cross-linked Tpm caused a significant decrease in its bending stiffness and a substantial reduction in the number of main chain H-bonds near the trypsin cleavage site of Tpm (Table 1 and Figure 3). The bending stiffness dropped to about half, while the number of H-bonds decreased by 37% (Table 1). In contrast, in the region of residues 129–138 in uncross-linked Tpm, the same increase in temperature did not induce any significant changes in bending stiffness or the number of the main chain H-bonds (Table 1 and Figure 3).

Despite the striking difference in the changes of bending stiffness of Tpm and the accessibility of its trypsin cleavage site upon an increase in temperature of cross-linked and reduced Tpm, the occupancy of the main chain H-bonds outside the cleavage site was insensitive to cross-linking (Table 1).

### 2.3. Interchain Cross-Linking did not Cause Convergence of Two Tpm α-Helices

**Figure 4:**
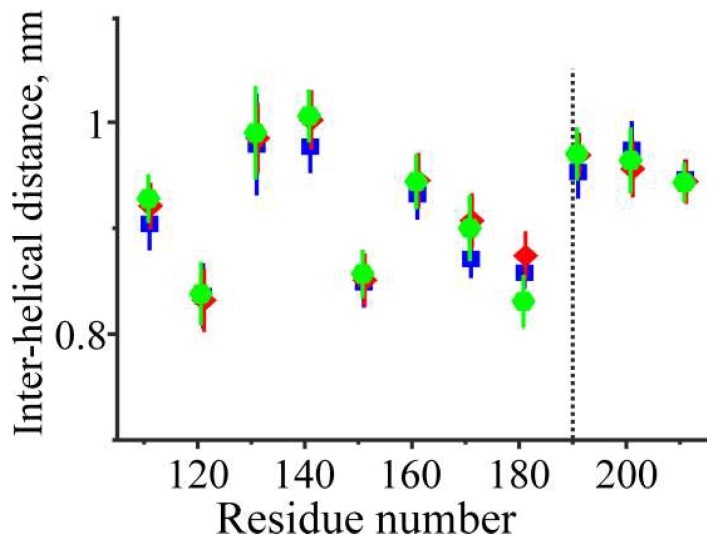
The distance between the two α-helices of the Tpm coiled-coil at different locations along the middle part of the Tpm molecule (mean ± S.D.) were obtained during MD simulation of three Tpm models at 27 °C. Blue squares, red diamonds and green hexagons correspond to uncross-linked, cross-linked and C190A Tpm, respectively. See Section 4 for further details of the analysis.

The time-average distances between the two α-helices of Tpm at different positions along its middle part obtained from MD simulations at 27 °C are shown in Figure 4.

Surprisingly, the formation of a disulfide bond between the Cys190 residues did not induce a local decrease in the distance between the two α-helices in the proximity of residues 190 (Figure 4). The tight coiled-coil structure was held without formation of a covalent bond for uncross-linked Tpm and even the C190A Tpm mutant where Cys residue was substituted for smaller Ala.

## 3. Discussion

### 3.1. Comparison with Experimental Data

The results of our MD simulations shown in Figure 2 and Table 1 agree well with the data from another recent report [22]. An increase in bending stiffness of the middle part of Tpm by 37–44% caused by the disulfide cross-linking (Figure 2 and Table 1) correlates with an increase in bending stiffness of the Tpm-actin filaments [22]. Besides, MD simulations did not show a significant difference between bending stiffness of the middle part of reduced Tpm and the C190A Tpm (Figure 2, Table 1). This result also agrees with experimental data showing that the bending stiffness of Tpm-actin filaments containing these Tpm constructs was similar [22,30].

The increase in the Tpm bending stiffness upon interchain cross-linking also correlates with an increase in the sliding velocity of reconstructed regulated thin filaments in vitro at saturating Ca^2+^ concentration [22]. A similar correlation was found previously for Tpm mutants containing different stabilizing mutations in the middle part of the molecule [25,29]. Heterogeneity of elastic properties of Tpm coiled-coil along its length revealed by our MD simulation (Figure 2 and Figure 3) correlates with heterogeneity of azimuthal movement of Tpm upon Ca^2+^ activation of thin filaments found by particle analysis of electron microscopy images [31].

### 3.2. Tpm Cross-Linking Decreases Bending Stiffness and Destabilizes the Trypsin Cleavage Site of Tpm at Elevated Temperature

The results of the comparison of structural properties of cross-linked and reduced Tpm in different experiments look somewhat controversial. The Tpm cross-linking stabilized calorimetric domain 2 that presumably includes the C-terminal part of Tpm [17,18]. Furthermore, it increased the overall bending stiffness of Tpm-actin filaments [22]. On the other hand, the cross-linking caused a decrease in the content of α-helices at temperature range 25–40 °C [15] and a “pre-transition” melting in the same range of temperature in DSC experiments [16,17]. This low temperature “pre-transition” was identified as the thermal unfolding of the least stable calorimetric domain 1 presumably corresponding to a region in the middle part of Tpm. These DSC data agree well with observations showing a ~4 °C shift (from ca. 46 °C to ca. 42 °C) of thermal dissociation of Tpm from actin upon cross-linking [22]. Besides, the cross-linking did not affect trypsin cleavage of Tpm at 26 °C, while this was accelerated at 40 °C [20]. Thus, it seems that the cross-linking simultaneously stabilizes and destabilizes Tpm.

These data can be readily explained by our MD simulations, which show a 37% decrease in the number of the main chain H-bonds in the region of the trypsin cleavage site at 40 °C, but not at 27 °C (Table 1). A sharp reduction in the number of main chain H-bonds near the trypsin cleavage site (Table 1) upon heating of cross-linked, but not reduced, Tpm explains the difference in the trypsin cleavage of cross-linked and uncross-linked Tpm at different temperature [20]. A significant decrease in the Tpm bending stiffness upon an increase in temperature from 27 to 40 °C of cross-linked, but not reduced Tpm (Figure 3, Table 1) may be responsible for a decrease in Tpm affinity for actin at higher temperature.

### 3.3. Possible Implications to the Pathogenesis of Heart Failure

Although in normally functioning hearts muscle Tpm is mainly in the reduced state [12], in failing human hearts [14] and in animals with hearts subjected to microembolization [13] or surgically induced myocardial infarction [32], the fraction of cross-linked Tpm increases. The results of our MD simulations together with recent [22] and earlier [15,16,17,18,19,20] experimental data allow one to speculate about a possible role of Tpm cross-linking in the pathogenesis of myocardial failure. A significant decrease in bending stiffness, a destabilization of the least stable part of Tpm at a temperature slightly above normal core body temperature (Figure 3, Table 1, [14,15,16,17,18,19,20]) and a decrease in Tpm affinity for actin upon Tpm cross-linking even at low temperature [21,22] can cause a partial loss of Tpm by thin filaments in cardiac myofibrils with cross-linked Tpm. This, in turn, could lead to incomplete myofibril relaxation and increased ATP consumption threatening with the diastolic failure of the heart and further escalation of the ischemic heart disease.

## 4. Materials and Methods

### 4.1. Structure Preparation

A crystal-based model of the middle part of the Tpm (Brown et al. [26], PDB code 2B9C), in which two Cys190 residues form a disulfide bond was used as the starting point for MD simulation of cross-linked Tpm. The models of uncross-linked Tpm and the C190A Tpm mutant were prepared and energy minimized using the CHIMERA package (CHIMERA was developed by the Resource for Biocomputing, Visualization, and Informatics at the University of California, San Francisco, CA, USA, supported by NIGMS P41-GM103311 [27]) and used as the starting point for their simulations.

### 4.2. MD Simulation

The MD simulations were performed for 100 ns using GROMACS v. 2016.3 package with 2 fs time steps [33]. The protein was immersed in a 9.5 nm × 9.5 nm × 24 nm rectangular box filled with water molecules extending at least 15 Å from the protein in each direction and periodic boundary conditions. The ionic strength of 0.15 M and zero net charge were provided by addition of Na^+^ and Cl^−^ ions.

After addition of water molecules, the system was energy minimized. First minimization was performed with the steepest-descent method with harmonic constraints applied to N, C_α_, and C backbone atoms until a maximum force of 1000 kJ·mol^−1^·nm^−1^ was achieved. Then energy was minimized further without any constraints and a maximum force of 100 kJ·mol^−1^·nm^−1^ was achieved. The system was then heated to 300 or 313 K during a 200 ps long MD run under an NVT (number of particles, volume, and temperature) ensemble with harmonically constrained C_α_ atoms (constraint stiffness of 4 kJ·mol^−1^·nm^−2^). The second 200 ps long preliminary MD run was carried out under NPT (number of particles, pressure, and temperature) ensemble using the Parrinello–Rahman barostat. The bond lengths were constrained with the LINCS algorithm [34]. The atomic coordinates of the systems were saved every 10 ps during each MD run for later analysis. The energy minimization and 100 ns long MD simulations were carried out using the AMBER99SB-ILDN force field [35] and the TIP3P water model [36].

### 4.3. Analysis of the MD Trajectories

The time course of the changes in the root mean squares deviation (RMSD) from the starting energy minimized structure was monitored [37]. We found that the equilibration was established within the first 10–20 ns of 100 ns long trajectories. The structured coiled-coil part of the middle part of Tpm was approximated by a polygonal line, as described previously [29]. Briefly, the centroids of the C_α_ atoms of 11-residue long segments (the 1st and 11th residues were taken with weight 0.5 to have about 3 turns of an α-helix in each segment) were found for each chain of Tpm in each snapshot (*j*-th time point). These segments contained residues 106–116, 116–126, etc. The last (11th) segment contained residues 206–216. Then, the midpoints between the two centroids were found and used as the vertexes of the polygon line approximating the coiled-coil at the *j*-th time point. The unit vectors ${\vec{t}}_{ij}$ parallel to vectors connecting consecutive *i*-th and (*i* + 1)-th vertexes in the *j*-th snapshot were considered as the unit tangent vectors of the coiled-coil at given positions along the coiled-coil at a given time point.

For an elastic bar subjected to Brownian forces (worm-like chain) the persistence length, *L*_p_, is proportional to bending stiffness, *k*: $L_{p}=\frac{k}{k_{B}T}$, where *k*_B_ is the Boltzmann constant and *T* is absolute temperature. For the intrinsically straight elastic bar, the time average scalar product of the tangent vectors $\vec{t}\left(s\right)$ and $\vec{t}\left(0\right)$ at the positions *s* and 0 along the bar axis is exponential of *s* [28]

(1) $$\langle (\vec{t}\left(s\right),\vec{t}\left(0\right))\rangle =exp\left(\frac{-s}{L_{p}}\right)$$

For an elastic bar that has an intrinsically curved shape, the expression can be generalized as follows [29]:

(2) $$\langle (\vec{t}\left(s\right),\langle \vec{t}(s)\rangle )\rangle =exp\left(\frac{-s}{L_{p}}\right)$$

where $\langle a\rangle =\frac{\sum_{j=1}^{N}a_{j}}{N}$ is the time average and $\langle \vec{t}\left(s\right)\rangle ={\vec{t}}_{i}=\frac{\sum_{j=1}^{N}t_{ij}}{\left|\sum_{j=1}^{N}t_{ij}\right|}$ is the time average unit tangent vector at the *i*-th position along the coiled-coil. Taking logarithms of both sides of this expression, one comes with a formula, $ln\left(\langle \left(\vec{t}\left(s\right),\langle \vec{t}\left(s\right)\rangle \right)\rangle \right)=\frac{-s}{L_{p}}$, convenient for the analysis of experimental data, as shown in Figure 2. The time averaging was performed for 8000 data points corresponding to the fragments of the MD trajectories from 20 to 100 ns.

The distances *d_ij_* between the centroids of the *i*-th segments of two Tpm α-helices in the *j*-th snapshot were considered as the distances between the helices at given location. The time average values $d_{i}=\langle d_{ij}\rangle$ and their standard deviations (S.D.) are shown in Figure 4 for all three Tpm structures studied here.

## Figures and Tables

**Table 1:** The persistence length *L*_p_ of the middle part of Tpm and the number of the main chain H-bonds estimated from MD simulations.

Tpm	t °C	*L_p_*^†^, nm	*L_p_* Ratio, XL/unXL ^†^	H-Bonds ^††^ Res. 106–129,138–160	H-Bonds ^††^ Res. 129–138	H-Bonds ^††^ Res. 161–215
unXL	27	98.4 ± 3.9 (110.8)	1(1)	73.4 ± 3.6	9.6 ± 1.4	81.8 ± 3.8
unXL	40	96.9 ± 4.7 (114.8)		70.3 ± 3.8	8.9 ± 1.7	80.0 ± 4.0
XL	27	141.7 ± 5.7 ** (151.6)	1.44(1.37)	72.4 ± 3.7	9.7 ± 1.3	78.6 ± 3.9
XL	40	74.5 ± 2.6 ** (80.5)		69.6 ± 4.0	6.1 ± 1.6 *	79.1 ± 4.1
C190A	27	106.3 ± 6.2 (114.8)	1.08(1.04)	71.1 ± 4.2	9.0 ± 1.7	81.5 ± 3.9

^†^*L*_p_ was estimated using the slope of either the least squares fit or secant line connecting the first and last points (in brackets). For details, see text and legend for Figure 2. ^††^ The number of the main chain H-bonds (Mean ± S.D.) in both Tpm chains at different locations. * The difference from the average number of main chain H-bonds in XL Tpm at 27 °C exceeds the sum of SDs. ^**^ Statistically significant difference from unXL Tpm at the same temperature (*p* < 0.01, Student’s *t*-test).

## Abbreviations

MD	Molecular Dynamics
Tpm	Tropomyosin
Tn	Troponin
XL	Cross-linked
H-bond	Hydrogen bond
S.D.	Standard deviation
PDB	Protein data base
S.E.M.	Standard error of mean

## References

[1] Perry S.V. (2001). Vertebrate tropomyosin: Distribution, properties and function. J. Muscle Res. Cell Motil..

[2] Nevzorov I.A., Levitsky D.I. (2011). Tropomyosin: Double helix from the protein world. Biochemistry.

[3] Behrmann E., Müller M., Penczek P.A., Mannherz H.G., Manstein D.J., Raunser S. (2012). Structure of the rigor actin–tropomyosin–myosin complex. Cell.

[4] McKillop D.F.A., Geeves M.A. (1993). Regulation of the interaction between actin and myosin subfragment 1: Evidence for three states of the thin filament. Biophys. J..

[5] Gordon A.M., Homsher E., Regnier M. (2000). Regulation of contraction in striated muscle. Physiol. Rev..

[6] Lehman W. (2016). Thin Filament Structure and the Steric Blocking Model. Compr. Physiol..

[7] Smith D.A., Maytum R., Geeves M.A. (2003). Cooperative regulation of myosin-actin interactions by a continuous flexible chain I: Actin-tropomyosin systems. Biophys. J..

[8] Smith D.A., Geeves M.A. (2003). Cooperative regulation of myosin-actin interactions by a continuous flexible chain II: Actin–tropomyosin–troponin and regulation by calcium. Biophys. J..

[9] Metalnikova N.A., Tsaturyan A.K. (2013). A mechanistic model of Ca regulation of thin filaments in cardiac muscle. Biophys. J..

[10] Lehrer S.S. (1975). Intramolecular crosslinking of tropomyosin via disulfide bond formation: Evidence for chain register. Proc. Natl. Acad. Sci. USA.

[11] Lehrer S.S., Ly S., Fuchs F. (2011). Tropomyosin is in a reduced state in rabbit psoas muscle. J. Muscle Res. Cell Motil..

[12] Lehrer S.S., Ly S., Fuchs F. (2011). Tropomyosin is in a reduced state in rat cardiac muscle. J. Muscle Res. Cell Motil..

[13] Canton M., Skyschally A., Menabo R., Boengler K., Gres P., Schulz R., Haude M., Erbel R., di Lisa F., Heusch G. (2006). Oxidative modification of tropomyosin and myocardial dysfunction following coronary microembolization. Eur. Heart J..

[14] Canton M., Menazza S., Sheeran F.L., Polverino de Laureto P., Di Lisa F., Pepe S. (2011). Oxidation of myofibrillar proteins in human heart failure. J. Am. Coll. Cardiol..

[15] Lehrer S.S. (1978). Effects of an interchain disulfide bond on tropomyosin structure: Intrinsic fluorescence and circular dichroism studies. J. Mol. Biol..

[16] Krishnan K.S., Brandts J.F., Lehrer S.S. (1978). Effects of an interchain disulfide bond on tropomyosin structure. Differential scanning calorimetry. FEBS Lett..

[17] Williams D.L., Swenson C.A. (1981). Tropomyosin stability: Assignment of thermally induced conformational transitions to separate regions of the molecule. Biochemistry.

[18] Kremneva E., Boussouf S., Nikolaeva O., Maytum R., Geeves M.A., Levitsky D.I. (2004). Effects of two familial hypertrophic cardiomyopathy mutations in α-tropomyosin, Asp175Asn and Glu180Gly, on the thermal unfolding of actin bound tropomyosin. Biophys. J..

[19] Matyushenko A.M., Artemova N.V., Sluchanko N.N., Levitsky D.I. (2015). Effects of two stabilizing substitutions, D137L and G126R, in the middle part of α-tropomyosin on the domain structure of its molecule. Biophys. Chem..

[20] Yampolsky D., Sumida J.P., Lehrer S.S. (2011). Effects of a disulfide crosslink (XL) on the trypsin cleavage pattern of rabbit cardiac tropomyosin (TM). Biophys. J..

[21] Walsh T.P., Wegner A. (1980). Effect of the state of oxidation of cysteine 190 of tropomyosin on the assembly of the actin-tropomyosin complex. Biochim. Biophys. Acta.

[22] Matyushenko A.M., Artemova N.V., Shchepkin D.V., Kopylova G.V., Nabiev S.R., Nikitina L.V., Levitsky D.I., Bershitsky S.Y. (2017). The interchain disulfide cross-linking of tropomyosin alters its regulatory properties and interaction with actin filament. Biochem. Biophys. Res. Commun..

[23] Williams D.L., Swenson C.A. (1982). Disulphide bridges in tropomyosin. Effect on ATPase activity of actomyosin. Eur. J. Biochem..

[24] Sumida J.P., Wu E., Lehrer S.S. (2008). Conserved Asp-137 imparts flexibility to tropomyosin and affects function. J. Biol. Chem..

[25] Matyushenko A.M., Artemova N.V., Shchepkin D.V., Kopylova G.V., Bershitsky S.Y., Tsaturyan A.K., Sluchanko N.N., Levitsky D.I. (2014). Structural and functional effects of two stabilizing substitutions, D137L and G126R, in the middle part of α-tropomyosin molecule. FEBS J..

[26] Brown J.H., Zhou Z., Reshetnikova L., Robinson H., Yammani R.D., Tobacman L.S., Cohen C. (2005). Structure of the mid-region of tropomyosin: Bending and binding sites for actin. Proc. Natl. Acad. Sci. USA.

[27] Pettersen E.F., Goddard T.D., Huang C.C., Couch G.S., Greenblatt D.M., Meng E.C., Ferrin T.E. (2004). UCSF Chimera—Visualization system for exploratory research and analysis. J. Comput. Chem..

[28] Li X.E., Lehman W., Fischer S. (2010). The relationship between curvature, flexibility and persistence length in the tropomyosin coiled-coil. J. Struct. Biol..

[29] Matyushenko A.M., Shchepkin D.V., Kopylova G.V., Bershitsky S.Y., Koubassova N.A., Tsaturyan A.K., Levitsky D.I. (2018). Functional role of the core gap in the middle part of tropomyosin. FEBS J..

[30] Nabiev S.R., Ovsyannikov D.A., Kopylova G.V., Shchepkin D.V., Matyushenko A.M., Koubassova N.A., Levitsky D.I., Tsaturyan A.K., Bershitsky S.Y. (2015). Stabilizing of the central part of tropomyosin increases bending stiffness of thin filament. Biophys. J..

[31] Paul D.M., Squire J.M., Morris E.P. (2017). Relaxed and active thin filament structures; a new structural basis for the regulatory mechanism. J. Struct. Biol..

[32] Avner B.S., Shioura K.M., Scruggs S.B., Grachoff M., Geenen D.L., Helseth D.L., Farjah M., Goldspink P.H., Solaro R.J. (2012). Myocardial infarction in mice alters sarcomeric function via post-translational protein modification. Mol. Cell Biochem..

[33] Abraham M.J., Murtola T., Schulz R., Pall S., Smith J.C., Hess B., Lindahl E. (2015). GROMACS: High performance molecular simulations through multi-level parallelism from laptops to supercomputers. SoftwareX.

[34] Hess B., Bekker H., Berendsen H.J.C., Fraaije J.G.E.M. (1997). LINCS: A linear constraint solver for molecular simulations. J. Comput. Chem..

[35] Lindorff-Larsen K., Piana S., Palmo K., Maragakis P., Klepeis J.L., Dror R.O., Shaw D.E. (2010). Improved side-chain torsion potentials for the Amber ff99SB protein force field. Proteins.

[36] Jorgensen W.L., Chandrasekhar J., Madura J.D., Impey R.W., Klein M.L. (1983). Comparison of simple potential functions for simulating liquid water. J. Chem. Phys..

[37] Zheng W., Barua B., Hitchcock-DeGregori S.E. (2013). Probing the flexibility of tropomyosin and its binding to filamentous actin using molecular dynamics simulations. Biophys. J..

